# A Review of the Evolution of Dairy Policies and Regulations in Rwanda and Its Implications on Inputs and Services Delivery

**DOI:** 10.3389/fvets.2021.611298

**Published:** 2021-07-23

**Authors:** Naphtal Habiyaremye, Emily Awuor Ouma, Nadhem Mtimet, Gideon Aiko Obare

**Affiliations:** ^1^Policies, Institutions, and Livelihoods, International Livestock Research Institute, Nairobi, Kenya; ^2^Department of Agricultural Economics and Agribusiness Management, Egerton University, Nakuru, Kenya; ^3^Policies, Institutions, and Livelihoods, International Livestock Research Institute, Kampala, Uganda; ^4^Strategy and Knowledge Department, International Fund for Agricultural Development, Cairo, Egypt

**Keywords:** dairy, policies, regulations, inputs, services, Rwanda

## Abstract

The dairy sector in Rwanda plays a key role in improving nutrition and generating income mostly for rural households. Despite the Rwandan 1994 genocide that left around 80% of dairy cows decimated, the dairy sector has experienced significant growth in the past two decades through government, development organisations, and donor programs, and through the nascent vibrant public–private partnership. In this paper, we reviewed and documented the evolution of the dairy policies, programs, and regulations in Rwanda and how they have contributed to the development of the dairy sector. The policy change has impacted the provision and use of inputs and services that have shaped the sector's milk production and productivity, milk quality, and demand. The results suggest that various policy- and program-level interventions have positively contributed to the growth of the dairy sector and improved the livelihoods of low-income households. This has been achieved through increased access to inputs and services, enhanced capacities of the public and private sector to deliver services, strengthened dairy cooperatives' governance, and increased value proposition to members of various farmer groups and promotion of milk consumption. We find that some of the implemented policies and programs, such as the “Girinka” (one cow per poor family) program, Rwanda Dairy Competitiveness Program II, and Rwanda Dairy Development Project, have resulted in improved farmer access to improved cow breeds and improved milk quality and cow productivity through enhanced health inputs and other services. While the dairy policies, programs, and regulations in Rwanda have paved the way for the development of the dairy sector and contributed to the provision and use of inputs and services, there are still challenges that need to be addressed. Accessibility and use of veterinary and artificial insemination services are limited by the quality of veterinary products, while the inadequate quality of feeds leads to low productivity of improved cow breeds. Consequently, farmers' uptake and use of inputs and services can be enhanced through a strengthened capacity of milk collection centres and health and animal feed policies that guide and control the quality of veterinary products and feeds sold in the markets.

## Introduction

The 1994 genocide heavily devastated the country's physical, economic, and social infrastructure, yet Rwanda experienced economic growth over the past two decades (1). This growth was led by an ambitious vision 2020, which was the country's long-term framework for development that sought to transform Rwanda into a middle-income country by 2020 (2). Although Rwanda did not achieve all its targeted goals of vision 2020, the country recorded an impressive gross domestic product (GDP) growth of 8% per annum (p.a) that led to an increase in GDP per capita from 211 to 718 USD between 2001 and 2014 and a poverty reduction from 59 to 39% (2, 3). Recognising the importance of the agricultural sector, the government of Rwanda (GoR) increased public investment in the sector and identified the sector as among the key drivers of vision 2020.

Over the past decade, the Rwandan agricultural sector grew at an average rate of 6% p.a (4). The sector plays a significant role in the economy of the country; it contributes about 31% of the total GDP and serves as the country's leading sector toward the achievement of the first and second Sustainable Development Goals (SDGs) of no poverty and zero hunger (2, 5). Furthermore, over two-thirds of Rwanda's labour force are employed in the agriculture sector, while more than 60% of the country's exports are from agriculture (6). Although various subsectors of agriculture have contributed to Rwanda's rapid aggregate growth, the dairy subsector is regarded as the fastest-growing subsector within agriculture as it contributes about 10.5% to the agriculture GDP (7).

Rwandan milk comes from cattle and goats. However, the dairy policies and interventions have been targeting milk from cattle as that from goats is negligible (8). In Rwanda, milk is consumed as raw, fermented (also commonly referred to as “Ikivuguto”), pasteurised, or processed products such as cheese, butter, ghee, and yoghurt (9). The country has three major dairy production systems, namely, zero grazing, open grazing, and semi-grazing (7, 10). Due to land resource scarcity in the country, zero grazing is the most common system in all regions where over 70% of production costs are related to feeds as cattle are kept in a shed and fed on forages. Open grazing is mostly found in Western and Northern highlands where cattle freely graze on individual or communal grazing lands. Semi-grazing is primarily practised in Eastern province, and it is characterised by a mixture of zero and open grazing where cattle are kept in stalls, fed on both forages, and grazed.

The GoR considers the dairy sector as a valuable pathway to economic growth. It not only contributes significantly to the country's total GDP but also offers a means of addressing malnutrition, famine, and poverty to the majority of cattle keepers and service providers along the dairy value chain (DVC) (11). In support of this dual function of the sector, the Rwandan government has been implementing different policies and regulations as well as partnering with various organisations aimed at initiating programs that improve the production and consumption of milk and increase incomes through livestock keeping. In this review, we consider the wide definition of policy by Anderson (12) as a “purposive course of action followed by an actor or set of actors,” which means that we consider not only the written government policies but also the actions and programs of various dairy stakeholders and DVC agents that lead to behavioural changes. Most policies and regulations were initiated to support government investments and programs that seek to transform the dairy sector from subsistence to a modern sector.

This paper documents the evolution of the dairy policies, programs, and regulations in Rwanda and assesses their contribution toward the development of the dairy sector, particularly in the provision and use of inputs and services that shaped the sector with regard to milk production and productivity, milk quality, and demand in the country. We also identify gaps that are not addressed by the current policies and the barriers to implementing specific regulations. The findings from this review will ultimately better inform dairy policy and decision making in Rwanda.

## Materials and Methods

This study comprised a literature review and key informant interviews. We reviewed journal articles, conference papers, reports, and “grey” literature. A wide internet search using search syntax such as [title: (dairy OR milk OR “dairy products”) AND (policy OR policies OR regulations OR program^*^ OR “dairy strategies” OR “dairy guidelines”) AND Rwanda] OR ab: (dairy OR milk OR “dairy products”) AND (policy OR policies OR regulations OR program^*^ OR “dairy strategies” OR “dairy guidelines”) was done. We also explored stakeholder websites, including the Ministry of Agriculture and Animal Resources (MINAGRI), Rwanda Agriculture Board (RAB), and Land O'Lakes. Other sites that provided important resources included Heifer International, International Fund for Agricultural Development (IFAD), International Livestock Research Institute (ILRI), and the Food and Agriculture Organisation of the United Nations (FAO). We reviewed 97 related documents, but we considered the information from 35 documents which include 19 journal papers, one book, seven project reports, and eight websites.

To get information on different policies and programs that were implemented, we conducted key informant interviews with 34 different dairy stakeholders in the country. Our key informants included one MINAGRI and two RAB staff, two staff members from Rwanda Agriculture and Livestock Inspection Services, one staff from Rwanda National Dairy Platform (RNDP), one staff from TechnoServe, one staff from Rwanda Dairy Development Project (RDDP), and a former staff of Rwanda Dairy Competitiveness Program II (RDCP II). Furthermore, our key informants included two board members and one manager from each of the seven Milk Collection Centres (MCCs) located in four different districts (Nyabihu, Ruhango, Rubavu, and Kamonyi) and three staff of an “inyange” milk processor as well as one staff of a milk retailer (fresh dairy kiosk) in Kigali. We also interviewed eight farmers from the four MCC districts to understand the effects of the initiated programs and six consumers to identify different types of milk available to consumers. All our interviews were conducted in-person while taking notes.

We qualitatively analysed this information and used the data from the Food and Agriculture Organisation Corporate Statistical Database (FAOSTAT) to provide a comprehensive image of the dairy sector in Rwanda. Our findings will serve as a basis for further grounded theory on dairy sector outcomes from policy interventions and complement the existing literature on the dairy sector development in Rwanda.

## Dairy Policies and Programs

### Girinka Program “One Cow Per Poor Family Program”

Over the past two decades, the GoR made important gains in rebuilding its livestock sector. After the 1994 genocide, around 90% of small ruminants and 80% of cattle were decimated, leaving the total cattle population at 162,683 in the country (7, 10). From 1995 to 2000, the cattle population started to increase as Rwandan refugees returning into the country came back with cattle. Dairy companies also started operations. In 2006, the GoR initiated the Girinka program, which means “One cow per poor family” to enhance social cohesion and improve family incomes, soil fertility, and nutrition. The Girinka program targeted the households in poverty who then received a dairy cow and were required to transfer the first calf to a qualified neighbour (13, 14). The households in poverty are usually identified using the “ubudehe” system, a comprehensive wealth-ranking system in Rwanda and is embedded into all administrative levels. Households are periodically ranked in their areas on a scale of 1 to 4 according to their poverty or wealth status (where category 1 is the poorest and category 4 is the richest) (7). For a household to benefit from the Girinka program, it must be in category 1 of ubudehe with the capacity to build a cowshed and holding land area between 0.3 ha and 0.75 ha (where 0.2 ha is allocated for cow feed) (13).

The Girinka program's rationale is to improve livelihood and increase nutrition among households in poor households through increased household income, milk consumption, and agricultural productivity (13, 15). It was expected that the given cow produces milk that is consumed by the household, generates income through milk sales, and produces manure that is used as fertiliser in crop fields. Considering that most cattle that were previously kept in Rwanda were indigenous or local breeds, the Girinka program distributed the pure breeds, consisting of mostly Friesian/Holstein and Jersey breeds. Despite the high feed ration demand of these breeds, they were, nevertheless, preferred due to their high milk production and that their progeny from crossbreeding with local cows is compatible with the local environment (13, 16).

The main agencies that have been implementing the program include the MINAGRI and non-government organisations such as Heifer International and Send a Cow. By 2015, around 203,000 households had received cows from the Girinka program, and these beneficiaries constantly receive services such as vaccinations, breeding, and advisory services from public veterinary personnel at subsidised costs (7, 14). Overall, the program has contributed to economic empowerment, poverty reduction, crop production, and improved nutritional status of beneficiary households (15, 17). Furthermore, the total cattle population increased from 645,848 to about 1,350,000 heads between 1997 and 2015, and the crossbreeds increased from 17 to 33%, while the pure breeds increased from 6 to 22% of total cattle between 2008 and 2015 (7, 18).

### Rwanda Dairy Competitiveness Programs I and II

The government's investments and efforts to support the dairy sector aroused different investors' and donors' interest in the sector in Rwanda. In 2007, the Rwanda Dairy Competitiveness Program I (RDCP I) was launched and implemented by Land O'Lakes International Development in collaboration with MINAGRI. The 4-year project that aimed at improving the competitiveness of the dairy sector in Rwanda, mostly targeting dairy farmers and the MCCs, ended in 2011 and was funded by United States Agency for International Development (USAID) (19). The project's “push” approach targeted the production side and strengthened the capacity of dairy farmers, giving more attention to farmers living with HIV/AIDS. It enhanced the profitability of dairy farms through increased milk production, improved milk quality at the MCCs, and enhanced the nutritional status of children in poor households and orphans by supporting the government's initiative of a school milk feeding program known as “One cup of milk per child.” Furthermore, the project trained about 3,500 farmers living with HIV/AIDS on cooperative management and animal husbandry and assisted in establishing a private Dairy Quality Assurance Laboratory (DQAL) that tests the quality of dairy products (19).

Despite the increase in milk production, the quality of the milk along the dairy value chain was still a concern. Therefore, to achieve the desired high-quality milk, Land O'Lakes, leveraging the momentum of RDCP I, implemented the Rwanda Dairy Competitiveness Program II (RDCP II). The RDCP II project was also funded by USAID and was implemented between 2012 and 2017 with the aim of improving the dairy competitiveness in the region, increasing milk production and consumption, as well as enhancing milk quality (20). The RDCP II was piloted in four milksheds (Northern, Southern, Eastern, and Kigali) covering 17 of the 30 districts of Rwanda. It was expected that quality milk that is produced efficiently and well-marketed throughout the entire value chain would improve the nutritional status of consumers and the income of smallholder producers (3).

**Figure 1:**
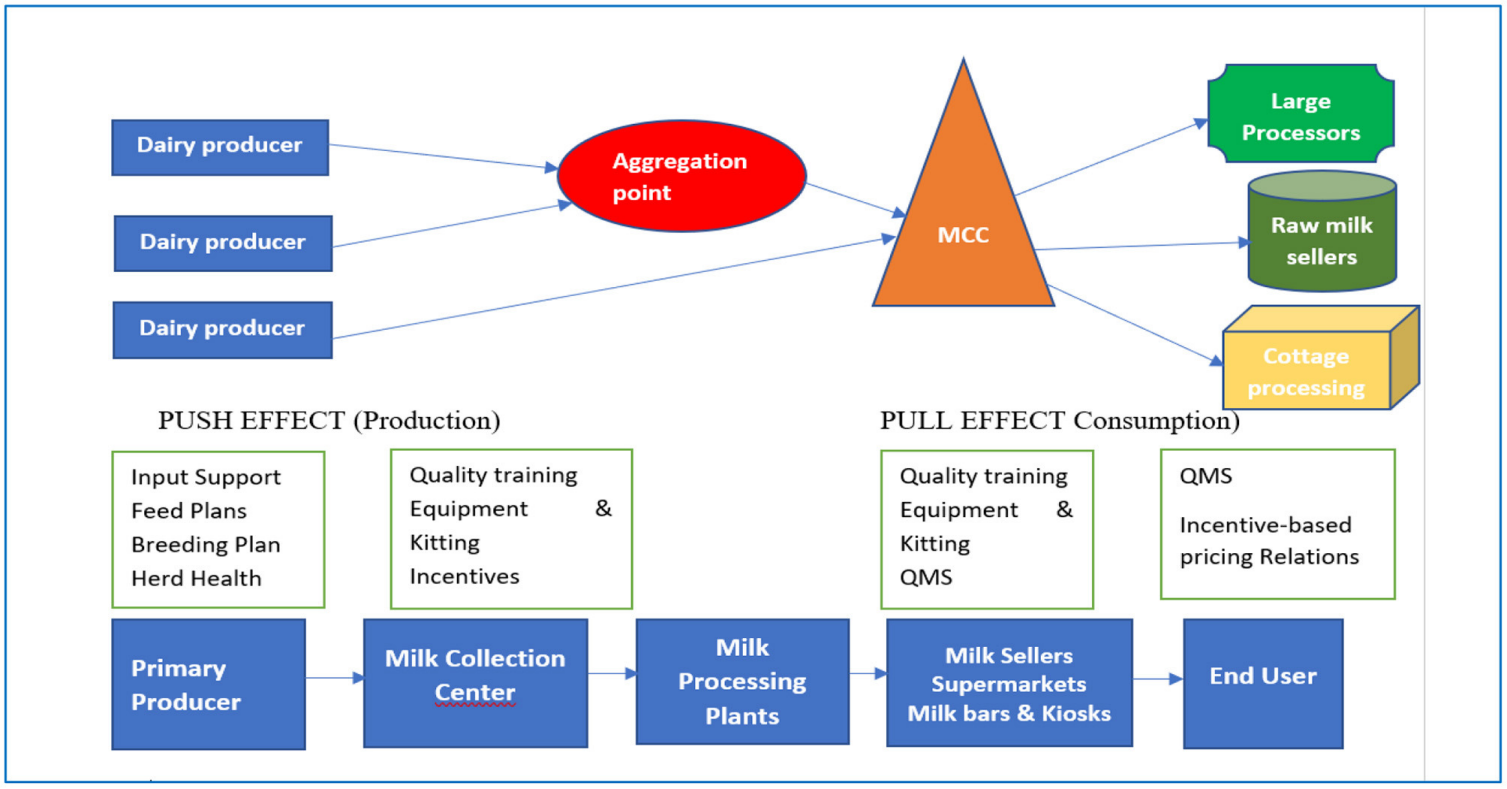
Schematic of the SOQ scheme. Source: Land O'Lakes (20).

In collaboration with MINAGRI, the RDCP II project initiated the dairy “seal of quality” (SOQ) certification scheme, which lays out a set of practises and standards for properly handling raw milk. The SOQ acts as an instrument for achieving the production and supply of quality milk. In this scheme, the dairy players that conform to the standards are given the SOQ certification that lasts for 12 months but is subject to renewal or withdrawal depending on the current compliance of the actors (20). The certification process is administered by the Rwanda Agriculture and Livestock Inspection Services (RALIS), a department under MINAGRI that issues the certificate to the MCCs and small processors who comply with the given standards. The awarded certificate is an intermediary stage that prepares those small processors to aim for the quality marks from the Rwanda Standards Board (RSB). Figure 1 presents the elements of the SOQ initiative.

The SOQ scheme at the farm level entails many processes that include: hygiene of the milker, cows and milk utensils, animal disease control and veterinary consultations, proper feeding of cows, and milk transport using stainless-steel cans. Furthermore, farmers are required to transport milk to an MCC or to an aggregation point where basic quality tests such as alcohol, lactometer, and organoleptic tests are conducted. The MCCs then distribute the milk to large processors, raw milk sellers, cottage cheese makers, and individual consumers. The milk quality inspection is done at the MCC and at the small processor levels, and it consists of an assessment of hygienic practises, mode of transportation, and milk cooling systems. In addition, a sample of milk is sent to a laboratory to test for somatic cell counts and bacterial counts.

The entry point of the RDCP II project was through the infrastructural improvement of dairy cooperatives and the MCCs in which they could reach out to the members. The project reached out to cooperative members through training in quality feed formulation, use of artificial insemination (AI), veterinary services, and milk handling practises (20). It also partnered with the Rwanda Council of Veterinary Doctors (RCVD) to train the AI technicians to enhance the accessibility and quality of AI services to farmers. The RDCP II encouraged the decentralisation of breeding technology and AI services through private service providers to enhance AI use in rural areas. Furthermore, RDCP II initiated a dialogue with different stakeholders and, in collaboration with RAB and the University of California, Davis, designed a strategic plan for national mastitis control that sought to reduce the occurrence of mastitis in the country (19, 20). In addition, MCC workers were trained on milk handling and quality, and the project supplied the MCCs with milk cooling tanks and milk testing kits, and it encouraged incentive-based pricing of milk using a milk grading system (20).

Upon the end of RDCP II, the MINAGRI changed the SOQ name to “Dairy Best Practise (DBP)” scheme to make it a national scheme and to distinguish it from the SOQ project-led scheme. However, the standards of the SOQ scheme and DBP scheme remain the same. Besides, in line with the policy pillar of the project, some dairy-related policies were implemented through the partnership of RDCP II, MINAGRI, and other stakeholders in the dairy sector. Some of the activities included the design of national dairy strategy (NDS), the creation of the Rwanda national dairy platform (RNDP), supporting the one cup of milk per child program, and a ministerial order to formalise the dairy sector.

### National Dairy Strategy

The NDS was a MINAGRI policy document designed and approved in 2013. It identified priorities and approaches to sustainably grow the dairy sector in Rwanda. The NDS was developed in consultation with stakeholders in the public and private sectors; hence, it was considered a roadmap to highlight possible barriers to developing the dairy sector and probable solutions (21). The NDS underlined the needed policies and strategies that would make the dairy sector competitive by providing affordable, accessible, and quality dairy products (21). Furthermore, the NDS emphasised the importance of public and private partnership (PPP) to achieve its objectives of improved production, stable marketing, and required policies that support the dairy sector.

The production objective of NDS was to increase milk productivity at the farm level while maintaining high-quality milk along the value chain. While the pure breeds from Girinka contributed to this, the GoR also invested in accessibility to AI and provision of animal health services and enhanced animal feed production during the dry and rainy seasons (7). This was done by promoting a public–private collaboration that requires private veterinarians and AI technicians to work closely with the MCCs. On the other hand, the marketing objective of NDS was to increase national milk consumption and to formalise the dairy value chain. Therefore, the government and RDCP II project created awareness on nutritional benefits of consuming milk among the population and boosted consumers' willingness to pay for processed milk instead of the unprocessed (20).

Various campaigns, such as *shisha wumva*, which means “feel the goodness” that used different strategies like radio slots, signs, and billboards, were launched, to drive behavioural change and create awareness of milk consumption in rural and urban areas (20, 22). These campaigns supported the already existing “One cup of milk per child” program that the government launched through RAB in 2010. The RAB program sought to address malnutrition among schoolchildren in districts with a high malnutrition rate. Over 83,000 pupils from 112 schools located in 15 districts were enrolled in this program where each child gets a half litre of milk twice a week (23). Furthermore, the government invested in improving rural roads and electrification as well as water supply and encouraged actors in DVC to improve milk value addition that expands milk marketing (7). Through the partnership of GoR and RDCP II, there was a renovation and establishment of new MCCs and dairy cooperatives to facilitate market access and enhance milk quality.

The policy side of NDS was aimed at attracting new investments in the dairy sector and initiating policies that support business transactions and competitiveness. The NDS proposed restructuring of the Rwanda National Dairy Board into the Rwanda National Dairy Platform (RNDP) as an inclusive organisation representing the interests of all dairy stakeholders (21). The RNDP was to ensure the implementation of the NDS and to advocate and promote the interests of all actors in DVC as it was formed based on a strong PPP (20, 21). Furthermore, the NDS sought to increase the trade of dairy products by proposing a harmonisation of tax and trade policies with those of Common Market for Eastern & Southern Africa (COMESA) and regional trade organisations. After meeting the COMESA standards, Rwanda's dairy trade improved, and the country is no longer a net importer of milk but also an exporter (4). While Rwanda has two main milk marketing channels (formal and informal), the NDS proposed a formalisation of the dairy value chain and due support for the SOQ program, which the government later backed through the issuance of a ministerial order (7, 21).

### The Ministerial Order

The GoR through MINAGRI issued the Ministerial Order (M.O) No. 001/11.30 of 10/02/2016 that stipulates the guidelines for collection, transportation, and selling of milk in Rwanda. The M.O supports the DBP certification by providing a set of procedures to farmers, milk transporters, MCCs, processors, and milk sellers and whose execution is to ensure that consumed milk is of high quality. The M.O requires that all milk leaving the farm gate should be collected at the MCCs where it is tested for quality prior to being sold. This means that the MCCs must have enough space, cooling tanks, and trained technicians and be equipped with milk quality testing equipment such as alcoholmeter, lacto-densimeter, thermometer, and antibiotic residue and mastitis test kits. Moreover, the M.O requires milk transporters to use well-closed stainless-steel cans or an appropriate vehicle with a cooling tank, while raw milk sellers are required to comply with the cleanliness of related utensils (24).

Despite the M.O's guidelines for formalising the dairy value chain, over 60% of milk is still sold through informal marketing channels in Rwanda (25). Generally, the informal marketing channel is characterised by an unorganised system where milk is not-industrially processed and sold directly to consumers in corner shops, in streets, from farmers, or from vendors, as well as door-to-door, which make the quality of milk questionable as the monitoring process and traceability are difficult (26, 27). Moreover, the informal milk marketing channel in Rwanda is the channel that does not follow the guidelines stipulated in the M.O, while the marketing channel follows the M.O's guidelines regulating the production, collection, transportation, and selling of milk (24). Conversely, the formal marketing channel is well-organised, characterised by legal licencing, and the milk sold in this channel is industrially pasteurised (26, 28).

While Doyle et al. (29) and Reeve (11) argue that the informal milk sector is associated with poor-quality milk potentially causing public health-related risks and diseases, there is a misperception that the milk sold in the informal sector is not automatically unsafe and the milk in the formal sector is not certainly safe (26, 28). This means that eliminating the informal sector based on quality achievement may negatively affect many poor households, mainly on the nutrition of infants and children (28). Therefore, it is prudent to identify the gaps that are yet to be addressed by the current policies and the barriers to implementing specific regulations.

### East African Dairy Development Project

The EADD project was a regional dairy sector development program whose phase 1 was implemented in Kenya, Rwanda, and Uganda from 2008 to 2013 and phase 2 was executed in Kenya, Tanzania, and Uganda from 2014 to 2018 (30). The project's aim was to lift farmers out of poverty through increased milk production and marketing (7, 30). The Bill and Melinda Gates Foundation funded the project, led by Heifer International in partnership with ILRI, TechnoServe, the African Breeders Service Total Cattle Management, and the World Agroforestry Centre.

The EADD project involved farmers and supported the initiation of milk hubs operated by dairy cooperatives, where farmers supply their milk for quality testing and chilling before it is sold (30). The project also linked the milk hubs with larger dairy companies and processors for stable milk markets. The EADD project supported dairy farmers in Rwanda by bringing the regional outlook in the country and providing training, and establishing MCCs as dairy hubs (7, 20). Besides the farmers' training on feed and cows' health improvement, the EADD project also trained local veterinarians on the provision of basic services such as vaccinations so that they are easily accessible at an affordable price (30). While the primary role of the MCCs is to provide a market and to ensure that the quality of milk is maintained, they also enhance farmers' access and use of inputs and services. For instance, through the inbuilt check-off system, farmers can access veterinary services and purchase feed supplements and milk cans from MCCs' stores at a lower price even when they do not have cash to pay for them as they are checked off against the milk supplied (30).

### Rwanda Dairy Development Project

The Rwanda Dairy Development Project (RDDP) is an ongoing project that was launched in 2016 to contribute to pro-poor economic growth and enhance the livelihood of poor rural households through dairy farming (7). The project seeks to promote climate-smart dairy farming practises and empower women and youth by integrating them into the dairy value chain (7). The project is funded by a concessional loan and grant from the International Fund for Agricultural Development (IFAD), private sector/banks, Heifer International, and the Rwandan government through tax exemptions. The RAB is the leading implementing agency in partnership with Heifer International, the Rwanda Cooperative Agency, the RNDP, the Business Development Foundation, and the Rwanda Council of Veterinary Doctors.

The RDDP has built on the past achievements in the dairy sector and is now concentrating on increasing cattle productivity, milk quality, and processing capacity of the dairy industry and strengthening the policy and institutional framework for the sector (7). This is done by improving farmer proximity to public and private animal health services reinforcing the capacities of public-sector veterinarians and establishing private sector-based networks, comprising animal health workers working under trained veterinary professionals. The RDDP is also focusing on strengthening dairy farmer cooperatives to efficiently provide services to farmers in the form of milk collection and payments and deliver dairy farming inputs to members through bulk purchases. It is also promoting the “hub model” that was successfully tested previously in other countries like Kenya, whereby the dairy cooperatives provide extension, AI, and animal health and financial services either directly or indirectly through linkages with the business development service providers, all geared toward a reduction in dairy market transaction costs (7).

The target of the RDDP is to meet the projected high domestic milk demand and maintain the upward trend in cross-border exports, mostly to the Democratic Republic of Congo and Burundi markets. Although the project is still ongoing, Taiwo et al. (31) found an increase in incomes of RDDP beneficiaries and improved access to extension services and credit facilities. Furthermore, the authors also found that the project has empowered many dairy hubs and dairy farmers' organisations and that, through the Livestock Farmer Field School approach, there has been an increase in the number of farmers able to access inputs and services such as AI, vaccinations, and improved forage seeds.

### Rwanda Livestock Master Plan

The Rwanda Livestock Master Plan (LMP) was developed in 2017 by ILRI, with substantial input from MINAGRI, RAB, and other research institutes and universities in Rwanda. Funding support was provided by the Food and Agriculture Organisation (FAO). It is assumed that the livestock sector will positively impact food and nutritional security in the country if the proposed investments are successfully implemented. The LMP is a series of 5-year investment plans for key livestock commodity value chains and production systems chosen based on priority development goals of the GoR. This document presents the visions, targets, challenges, and policy required to achieve the expected outcomes in the government's priority value chains, which include cow dairy, red meat, poultry, and pork (8). The Rwanda LMP is considered as a guiding document to policymakers and all agents engaged in livestock development. The priority investment interventions are meant to meet the agreed national goals, including poverty reduction, achieving food security, increasing economic growth and exports, contributing to industrialisation and employment, and mitigating greenhouse gas (GHG) emissions (8).

To increase milk production to meet the projected increased domestic demand and surplus for export, the LMP presents the dairy value chain development roadmap of 2017/18 to 2021/22. To achieve this, the plan highlights priority interventions in feeds and feeding, animal health, extension services, genetics, processing, and marketing. It also identifies livestock feeds, as the main challenge toward improving livestock productivity and particularly cattle farming (8). Therefore, the LMP proposes the promotion of improved grass and leguminous feed productions in all available areas such as backyards, hedges, and fences. It also recommends creating an industry that produces feed additives and allocation of land for production of improved forage and promotion of the use of concentrates or processed feeds (8).

The priority intervention in animal health highlighted in the LMP is to address the insufficiently trained veterinary personnel and the prevalence of mastitis. Over 60% of cattle in the country have mastitis cases (8). Therefore, the plan seeks to support veterinary diagnosis laboratories, enhance veterinary coverage through PPP, and reinforce disease surveillance and mass vaccination programs' capacity. It projects that by 2021/22, Rwanda will be free from foot and mouth disease (FMD) and contagious bovine pleuropneumonia (CBPP) (8). Furthermore, LMP plans to make vaccines accessible and projects that around 60% of farmers will have adopted mastitis control and management technologies and the recommended rate of tick control treatments by the year 2021/22 (8). Furthermore, the LMP recommends building the capacity of extension agents, providing intensive farmers' training on dairy improvement, and increasing extension service delivery through producer organisations.

Cattle genetics is also the priority intervention in Rwanda LMP where the target is to reduce the local breed while increasing the number of crossbreeds and pure breeds. While the number of local breeds decreased annually at a rate of 4% in the past decade, the LMP's goal is to increase crossbreed cattle by a rate of 8% annually by the year 2021/22 (8). Considering that in 2016/17, only 15% of cows were getting AI services, the training of AI technicians and the promotion of private AI practitioners to make AI service more accessible to rural communities were among recommendations of the LMP. On processing and marketing priority interventions, the plan sets some ambitious goals of establishing around 150 MCCs, 200 milk collection points (MCP), and 150 dairy cooperatives while strengthening the existing ones to fully comply with milk quality standards found in the M.O (8). Moreover, the LMP aims to attain a functional linkage between private milk traders, MCCs, cooperatives, and processing plants so that milk price is based on quality. In addition, the LMP seeks to improve feeder roads to and from the MCCs and enforce the M.O so that around 80% of milk is sold in formal market. These will not only incentivize the establishment of new processing plants but also increase the attraction of local and international investors in Rwandan DVC (8).

## Discussion

**Figure 2:**
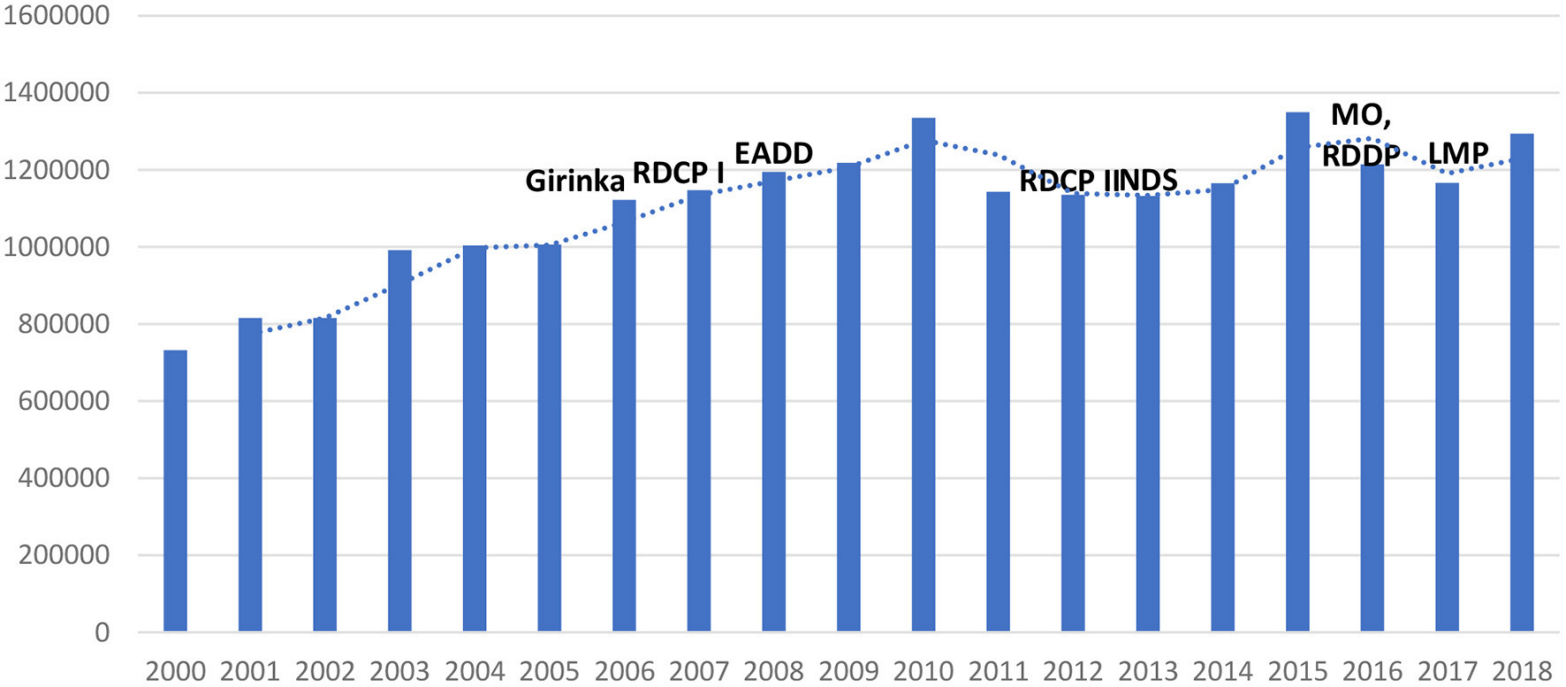
Total number of cattle in Rwanda over time. Source: Based on FAO data (FAOSTAT: http://www.fao.org/faostat/en/#data/QA).

**Figure 3:**
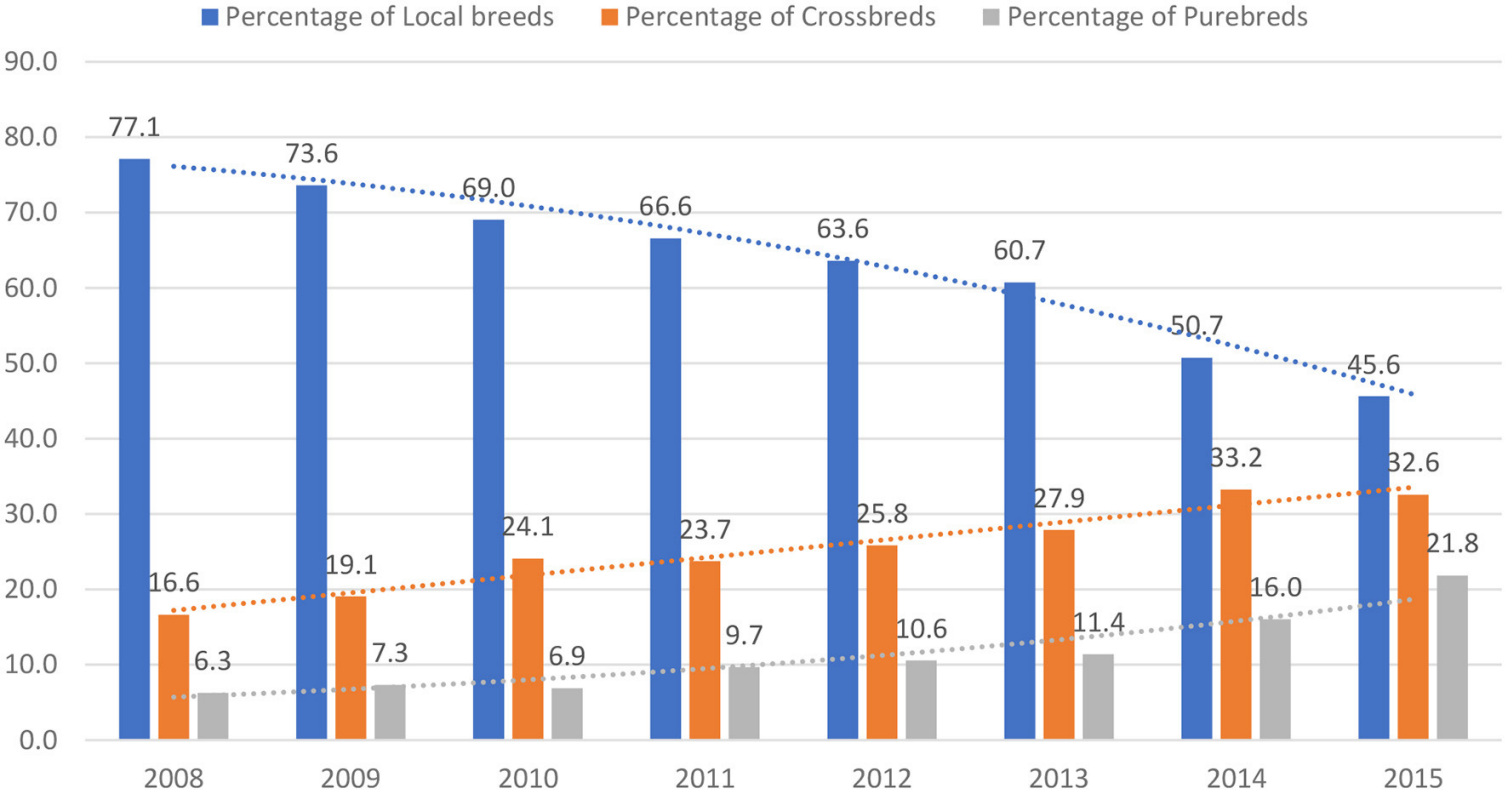
Percentage of cattle breeds in Rwanda between 2008 and 2015. Source: Based on data from IFAD (7).

**Figure 4:**
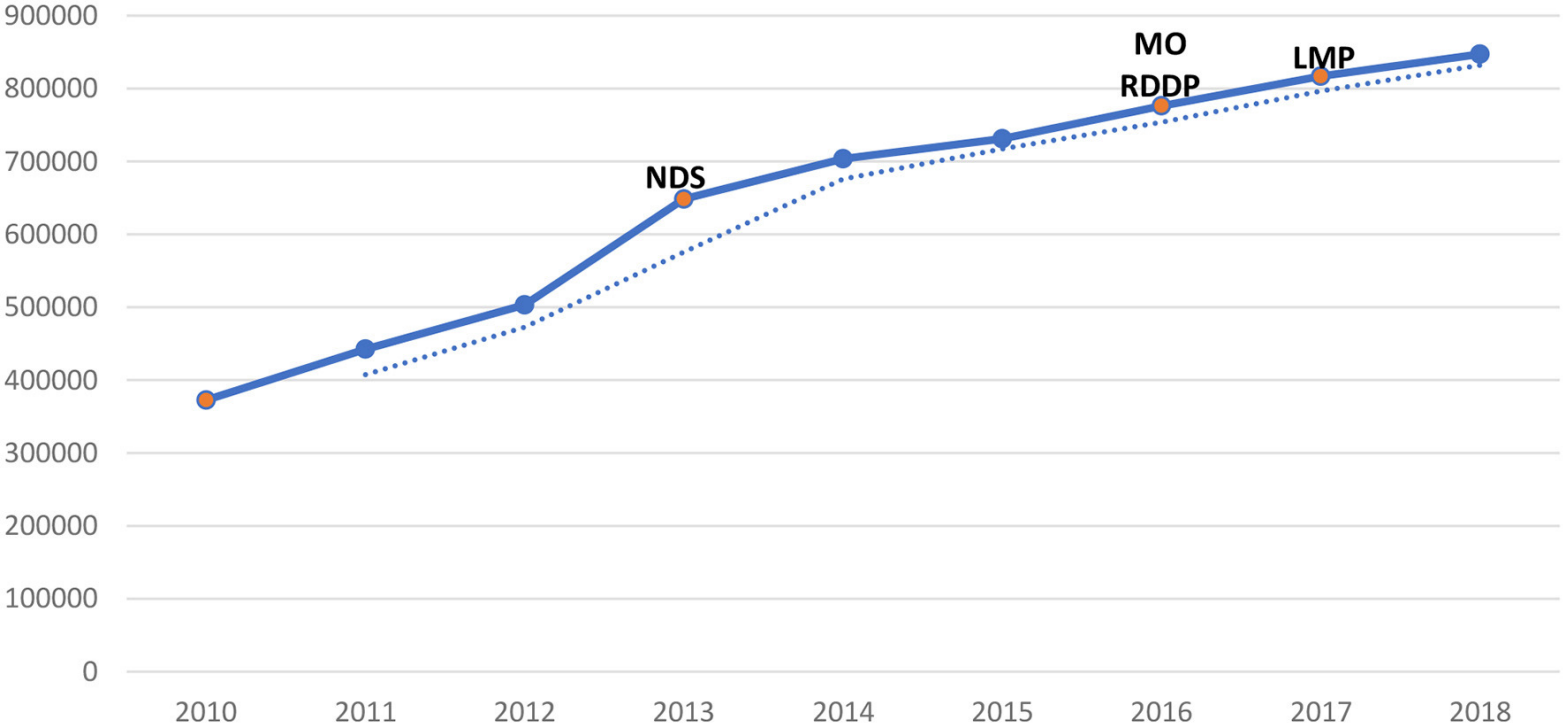
Cow milk production trend in Rwanda in metric tonnes (MT). Source: Based on data from MINAGRI annual report (4).

**Figure 5:**
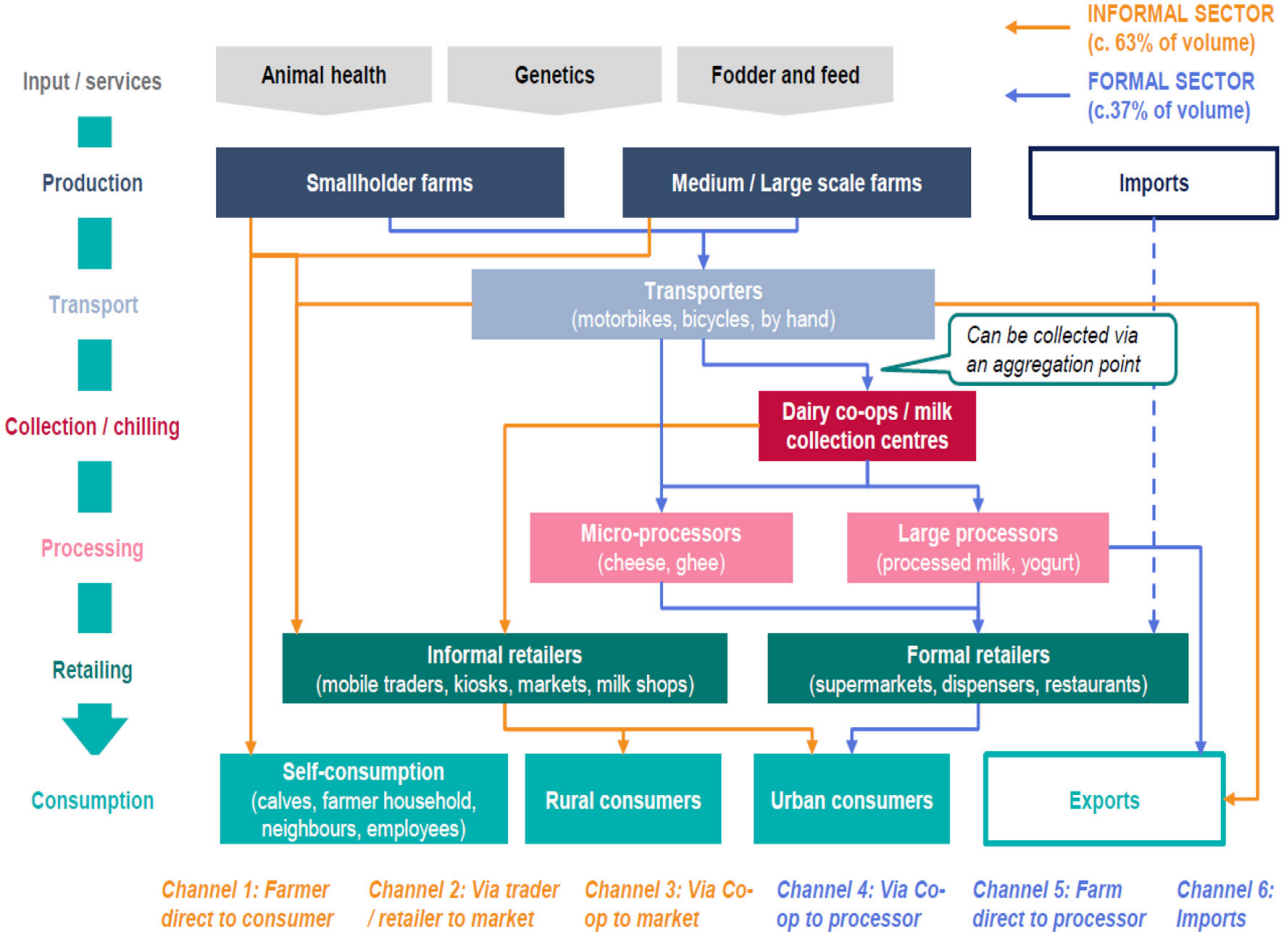
Dairy value chain in Rwanda. Source: TechnoServe (10).

For the past two decades, several dairy policies, regulations, and programs have been implemented in Rwanda with the aim of improving and promoting the dairy sector, as discussed in the previous sections. Investments in the dairy sector have become financially viable as long as farmers and other DVC actors follow the dairy best practises (32). Undoubtedly, these policies and programs have increased farmers' access and use of different inputs and services, leading to the growth of the dairy sector in the country. Some of the subsequent effects include an increase in cattle population (Figure 2), a shift from local breeds to crossbreeds and pure breeds of cattle (Figure 3), and enhanced dairy cow productivity in the form of milk volume (Figure 4). Furthermore, the dairy sector has been well-shaped as a result of improving different agents of the value chain (Figure 5).

An analysis of the FAOSTAT data shows that the total cattle population in Rwanda has increased in the past two decades from about 732,000 in 2000 to ~1.3 million in 2018 (Figure 2). There was a decrease in total cattle population between 2015 and 2017 caused by cattle mortality due to diseases such as tick-borne diseases and Rift Valley fever (RVF) and a prolonged drought experienced during that period (5). Our key informant farmers, who are Girinka program beneficiaries, confirmed that receiving a cow has not only given them access to milk which they were previously unable to purchase, but they also earned some income from milk sales.

Conversely, Figure 3 shows a significant shift from local cattle breeds to crossbreeds and pure breeds because of the Girinka program implementation and investments in AI services. In 2008, the local breeds represented 77% of the total cattle population in Rwanda, but by the year 2015, the crossbreeds and pure breeds were 33 and 22% of total cattle, respectively. Our interviews with farmers confirmed that every farmer is striving to get a crossbreed or a pure-breed cow. Farmers express their preference for improved breeds due to their high productivity, longer lactation length, and shorter calving interval. Moreover, those farmers with sufficient finances prefer to buy the crossbreeds or pure breeds, while those with inadequate money use AI or purebred bulls until they get an improved-breed calf.

The interviews with key informants from RAB and MINAGRI attributed the increased milk production to the increase in cattle population and the gradual shift from local breeds to crossbreeds and pure breeds. They argue that crossbred and purebred cows have a higher productivity compared to local breeds when properly fed and if appropriate animal husbandry practises are followed. The MINAGRI annual report of 2018/19 shows that milk production has more than doubled between 2010 and 2018, and milk consumption has increased from 37.3 l per capita in 2010 to 69.4 l per capita in 2018 (4). Although milk consumption per capita is still below the World Health Organisation (WHO)-recommended 220 l per capita per year, the LMP aims to achieve this level by the year 2031/32 (8). Figure 4 shows a general increase in milk production in Rwanda between 2010 and 2018.

The productivity gains on milk and manure production as well as on improved animal health were realised. Miklyaev et al. (32) found that daily milk production doubled from 5 to 10 l per cow, which led to an annual increase of milk yield per cow from 608 to 1,949 l in RDCP II coverage areas. It was also established that there was a decrease in the calving interval from 18 to 15 months, a 2-fold manure production at farm level, and a drop in calf mortality from 15 to 10% due to increased feed and adoption of animal health services. Our interviews with RAB and MINAGRI staff corroborate these findings, although they recognised the gap in milk productivity as improved breeds are producing below their potential. They attributed the low productivity to farmers' lack of proper cow management, such as insufficient and/or imbalanced feeds and inappropriate animal husbandry practises.

Increased milk production was realised together with improved milk quality along the DVC, which has enabled the sector to become competitive regionally by meeting the COMESA quality standards (3, 20). The interview with RALIS staff and the MCC key Board Members confirmed that many MCCs have been working with farmers to comply with quality requirements, an element that has reduced the quantity of milk rejected at the MCCs. Whereas, Rwanda has been a net importer of milk, the increased milk production and improved milk quality enabled the country to export surplus milk. In 2018, the country imported 0.118 MT of milk products such as powdered milk and butter, while it formally exported about 4 million litres of pasteurised milk and 1.5 million litres of UHT milk (4). In addition, informal milk exports to Burundi and the Democratic Republic of Congo (DRC) were estimated to be around 15 million litres annually (7). Furthermore, the SoQ expanded the business opportunities to milk agents through existing milk products such as cheese, butter, and ghee that are both consumed locally and exported (20). The Rwanda LMP aims at a 46% increase of crossbred dairy cattle, 65% increase of milk production, and 41% increase of cattle productivity under the recommended level of investment scenario (8). If these targets are achieved, then further policy outcomes will be realised by 2021/22.

While there has been a progressive shift from local cattle breeds to crossbreeds and pure breeds, the interviewed farmers are concerned about the availability of feeds required to ensure consistency of milk supply, especially during the dry season when feeds are insufficient. This is because improved breeds may not attain their potential productivity if they are not fed on balanced feed rations. The implemented interventions have enhanced training on technologies related to conservation of forages for dry seasons, incorporating crop residues and crop by-products as feeds, establishing feed processing plants, and feeding on complementary feed sources (7, 20). Our interview with former RDCP II staff confirmed that the project promoted feed conservation technology such as making silage and cultivation of legumes. However, the MCC board members are worried about the sustainability of these interventions as they require strong support from the private sector to ensure that these inputs are accessible to farmers.

To facilitate milk marketing and processing, the dairy sector in Rwanda was divided into five milksheds, namely, Eastern, Western, Southern, Northern, and Kigali (7). Each milkshed has a big processor responsible for collecting and buying milk from MCCs located in that geographical area. Besides, the MCCs have been empowered through leadership, governance and management training, and enhanced storage capacity. Furthermore, the compliance to the M.O has increased the volumes of milk supplied to the MCCs, which further improved the formal milk marketing channel (20). Despite the role of the milkshed system in providing markets by linking MCCs to processors, it is also disadvantageous to farmers as it limits competition among buyers. This is because processors are only allowed to buy milk from their milkshed. Thus, this system is more beneficial to processors as they buy milk from the MCCs at a low price while the price farmers sell to the MCCs depends on the price the MCCs receive from the processors.

Although farmers are encouraged to adopt better farming practises, farm-gate milk prices are relatively low, where the farmers' share of the final consumer price of milk is 16% compared to international standards of 50% (21). Packaging costs and limited competition among processors are the main contributors to the high price of processed milk (10). Policies geared toward reducing production costs at the upstream channel, including packaging, would reduce the margins between the consumer and producer prices to the advantage of both market participants. At the same time, an expansion of marketing options within milksheds will improve competition from the demand side. Although the “Inyange” processor has invested in milk zones that sell fresh pasteurised but unpackaged milk at an affordable price (20), this system can be upscaled to all districts to easily make this type of milk accessible to the majority of consumers, especially in peri-urban and rural areas. This can be done by introducing milk-dispensing machines (or milk ATMs) as it is the case in Kenya, which require less infrastructure and human resource than milk zones. Figure 5 below presents the current dairy value chain in Rwanda.

While the dairy sector may be vulnerable to climate change on both the production and marketing sides, it may also contribute to climate change as an increase in dairy production may lead to high GHG emissions if better dairy management practises are not used. Grewer et al. (3) analysed and estimated the effects of RDCP II on GHG emission intensification using the FAO Ex-Ante Carbon Balance Tool (EX-ACT). They found that RDCP II contributed to a reduction of GHG emission intensity (in the project area) by −4.11 tCO_2_e per 1,000 l of milk (−60%) and −1.7 tCO_2_e per 1,000 l (−47%) in extensive and intensive production systems, respectively. This was achieved through improved feed quality and quantity, herd weights, herd size management, and breeding services (3). Herrero et al. (33) found that low-quality feeds may lead to reasonably high GHG emissions from enteric fermentation per unit of meat or milk due to its low digestibility.

It thus follows that feeding quality forage-based diets supplemented with concentrates and agro-industrial by-products would lead to higher milk production per cow, hence lowering GHG emission per unit of milk produced (34). Similarly, improved animal health and breeding services such as the use of AI decrease GHG emission levels through reduced herd overhead (33, 34). It is expected that Rwanda dairy policies will further contribute to a reduction of GHG emissions as mitigating the contribution of livestock to GHG emissions is one of the Rwanda LMP objectives (8). Moreover, the ongoing RDDP promotes climate-smart dairy farming (7).

## Conclusion and Recommendations

The dairy policies, programs, and regulations in Rwanda have led to an improved dairy sector in the country and contributed to the provision and use of inputs and services. Some of the policies and programs that have been implemented, such as Girinka, RDCP I and II, and RDDP, have enhanced dairy productivity, input market, and milk production through enhanced health inputs and other services. Despite the remarkable growth of the Rwandan dairy sector, the sector still lags behind those of other countries in the region, such as Kenya and Uganda, in terms of milk productivity and consumption (10). There are still some challenges in the dairy sector and barriers to implementing specific regulations. These include the quality of veterinary and AI services, insufficient human resource capacity, low productivity of crossbreeds and pure breeds, insufficient and inadequate quality of feeds, limited competition among milk buyers, informal marketing channels, and insufficient number of MCCs. This calls for strategic investments and more in-depth research that would lead to the formulation of evidence-based policies.

Whereas, accessibility and use of veterinary and AI services have improved, they are still limited by the quality of veterinary products, inadequate human resource capacity, and semen scarcity, while the insufficient and inadequate quality of feeds contributes to low productivity of crossbreeds and pure breeds (9). More policy-driven responses in terms of access to semen and enhancing the number of bull stations are needed, along with health and animal feed policies that guide and control the quality of veterinary products and feeds sold in the markets. It is recommended that a strong PPP that provides adequate youth training on veterinary services, as well as AI technicians to improve farmers' access and use of inputs and services, be initiated and promoted. Furthermore, policies that promote legumes and grass conservation would boost the availability of enough feed from the same land allocated to feed cultivation.

While the MCCs make inputs and services accessible to farmers, the primary concern is that they are still insufficient, and not all established MCCs are well-functioning (7). Therefore, there is a need for designing and implementing policies that provide incentives to the private sector to invest in the establishment of the MCCs across the country and improve their capacity so that farmers can easily access and use the inputs and services. Also, there is a challenge in the transitioning of local breeds to pure breeds or crossbreeds as local breeds still represent 43% of the total cattle population while they only contribute 9% of total milk production in the country (8). Interventions geared toward enhancing the gradual reduction of local dairy cows with improved breeds combined with better management and animal husbandry practises would address the negative correlation between milk production and the number of cattle.

Any policy intervention that seeks to eliminate the informal sector completely may not be successful as it happened in Kenya 10 years ago. Given the failure of the policy, Kenya chose to integrate informal market traders through a training and certification scheme, which ended up improving the quality of milk in the informal sector (28, 35). Incorporating the informal marketing channel in dairy policy formulation rather than its elimination would improve the dairy sector in Rwanda, and other developing countries, where the informal sector is more dominant. This can be done by training and integrating informal milk traders and middlemen to test the milk before they collect it from the farmers as it is the case in the formal sector.

Credible evidence is relevant in lieu of any policy changes. Leksmono et al. (36) highlight the role of research in developing the dairy policy. They found that policy change can easily be realised when the focus is first made on research and development rather than on policy formulation. Therefore, appropriate marketing research may lead to evidence-based policy that accommodates and improves the informal marketing channel. Conducting research on breeds' productivity under different environments would be a useful input to a national breed policy while farmers' adoption of research-based improved forages will address the low productivity of crossbreeds and pure breeds. This study recommends that further farm-level studies are conducted to assess the profitability of better dairy farming practises, given the current policies and more research on dairy projects before dairy policies and programs are initiated.

## Author Contributions

NH contributed to the conception, design, literature review, conducting of key informant interviews, interpretation of the findings, and drafting of the manuscript. EO, NM, and GO contributed to the conception, design, and interpretation of the findings and drafting and substantive revision of the manuscript. I confirm that all the authors have contributed significantly in the generation of this manuscript.

## Conflict of Interest

The authors declare that the research was conducted in the absence of any commercial or financial relationships that could be construed as a potential conflict of interest.

## Publisher's Note

All claims expressed in this article are solely those of the authors and do not necessarily represent those of their affiliated organizations, or those of the publisher, the editors and the reviewers. Any product that may be evaluated in this article, or claim that may be made by its manufacturer, is not guaranteed or endorsed by the publisher.

## Abbreviations

AI: artificial insemination
DBP: dairy best practises
DVC: dairy value chain
FAO: Food and Agriculture Organisation of the United Nations
GDP: gross domestic product
GHG: greenhouse gas emission
GoR: Government of Rwanda
IFAD: International Fund for Agricultural Development
ILRI: International Livestock Research Institute
LMP: Livestock Master Plan
MCC: Milk Collection Centre
MINAGRI: Ministry of Agriculture and Animal Resources
MO: Ministerial Order
NDS: National Dairy Strategy
PPP: public–private partnership
RAB: Rwanda Agriculture Board
RDCP: Rwanda Dairy Competitiveness Program
RDDP: Rwanda Dairy Development Project
RNDP: Rwanda National Dairy Platform
SOQ: seal of quality
USAID: United States Agency for International Development.
